
JOURNAL INFORMATION
==============================
NLM Title Abbreviation: ScientificWorldJournal
Journal ID: ScientificWorldJournal
NLM Journal ID: 101131163
Journal ID: TSWJ

The Scientific World Journal

ISSN: 2356-6140
EISSN: 1537-744X
Publisher: Wiley

ARTICLE INFORMATION
==============================
PMCID: PMC6084033
PMID: 30147493
DOI: 10.1100/tsw.2001.149
Article ID: 261481
Article version: 1
Subjects: Short Report

Establishment of Okadaic Acid Resistant Cell Clones using CDNA Library Expression Cloning

Sandal Tone *
Lillehaug Johan
D skeland Stein Ove
Ahlgren Ragnhild
Department of Anatomy and Cell Biology, University of Bergen, Årstadveien 19, N-5009 Bergen, Norway
*Tone Sandal: tone.sandal@iac.uib.no
Publication date: 2001
Electronic publication date: 2001 Oct 23
Volume: 1
First page: 42
Last page: 42
Copyright: Copyright © 2001 Tone Sandal et al.
Copyright year: 2001
License: This is an open access article distributed under the Creative Commons Attribution License, which permits unrestricted use, distribution, and reproduction in any medium, provided the original work is properly cited.
License URL: https://creativecommons.org/licenses/by/3.0/

Funding: Norwegian Cancer Society

==============================
Miami *Nature Biotechnology* Short Reports
*TheScientificWorld* (2001) 1(S3), 42SR
ISSN 1532-2246; DOI 10.1100/tsw.2001.149

### ESTABLISHMENT OF OKADAIC ACID RESISTANT CELL CLONES USING CDNA LIBRARY EXPRESSION CLONING

*Tone Sandal, Johan Lillehaug, Stein Ove Døskeland, Ragnhild Ahlgren

*Department of Anatomy and Cell Biology, University of Bergen, Årstadveien 19, N-5009 Bergen, Norway

- tone.sandal@iac.uib.no

**INTRODUCTION.** The broad action of the phosphatases inhibited by this universal apoptogen has made it difficult to pinpoint the relevant molecular targets, and mechanisms involved in OA induced apoptosis(1,2,3,4). Retroviral cDNA-library expression cloning technique (5) report the isolation of more than twenty OA resistant cell clones, and characterisation of two of them reveals that the approach can produce OA resistant cell clones to be used as important tools to investigate mechanisms of apoptosis induced by serine/threonine phosphatase inhibitors.

**METHODS.** A Jurkat T-cell cDNA library, constructed in the retrovirus vector pBabeMN, was applied to produce recombinant retrovirus, according to previously described procedures (6) Mouse embryonic fibroblasts, C3H/10T1/2-cl-8. The bidirectionally oriented cDNA library, containing 1 x 106 independent cDNA inserts, was a kind gift from Dr. Nolan, Stanford University. After three rounds of screening, with lethal doses of OA, on infected cells, surviving OA resistant cell clones were selected. Isolation of cDNA from selected cell clones was performed by RT-PCR, characterised by sequencing and their antiapoptotic effect confirmed by recloning and retransfection into parental cells.

**RESULTS.** Twenty-one OA-resistant C3H10T1/2-cl-8 cell clones were selected after infection with a retroviral JurkatT-cell cDNA expression library (Fig. 1). Those clones with highest OA-resistance upon re-challenge (OAR-1, OAR-2) had retrovirally introduced, short cDNAs corresponding to two human genes with unknown function (Fig. 2). The *oar1*-cDNA sequence, in the antisense orientation, is nearly identical to a human DNA sequence (AP000795) localised on human chromosome 11 (11q14), recently submitted to the database. It is therefore most probable that *oar1* acts as antisense RNA against mRNA coded by AP000795 or a similar gene not yet reported.

**Fig. 1**

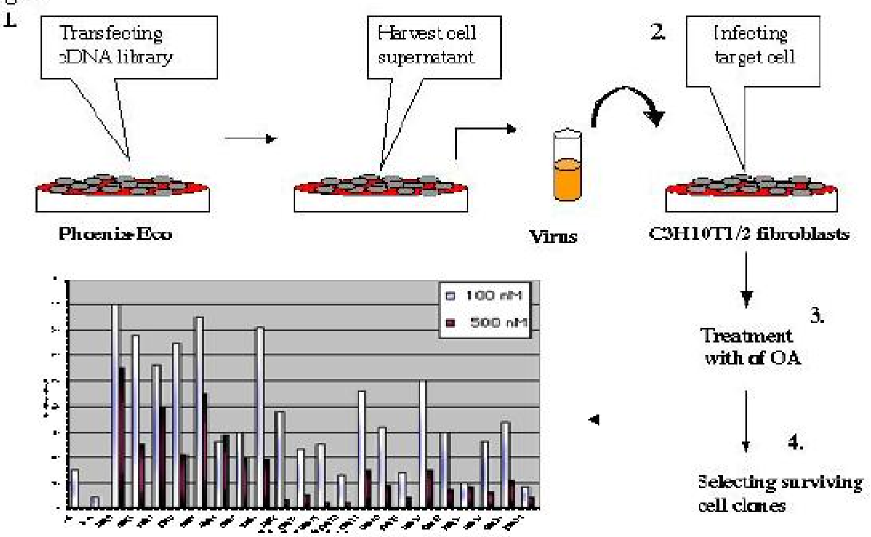

figure 1

*Oar2* is nearly identical to human DNA sequences within a gene (AK002158) with unknown function. *Oar2* appears to have one region (nucleotides 226-376) that is identical to the predicted last part of the reading frame of AK0021258. The rest of *oar2* corresponds, in antisense direction, to sequences within the 3ÚTR of AK0021258. There is a potential large open reading frame including part of the antisense sequence and the whole of the sense sequence.

Reintroduction of *0ar-1* or *0ar-2* into wild-type cells reproduced the OA-resistant phenotype. OAR-1,2 were cross-resistant to other phosphatase inhibitors, but fully susceptible to staurosporin, indicating selective resistance to phosphatase inhibitors. The defect(s) in OAR1,2 appear to be downstream of phosphatases, since the 2-D phosphoprotein pattern was similarly affected by OA in wt cells and OAR1,2. We found that OA induced cell death was partially protected by the caspase inhibitor zVAD-fmk in both wt, OAR1 and OAR2, and neither clone was protected against microinjected cytochrome c, indicating that the defects are upstream of or independent of zVAD sensitive caspases and the apoptosome (apaf-1/caspase 9/3).

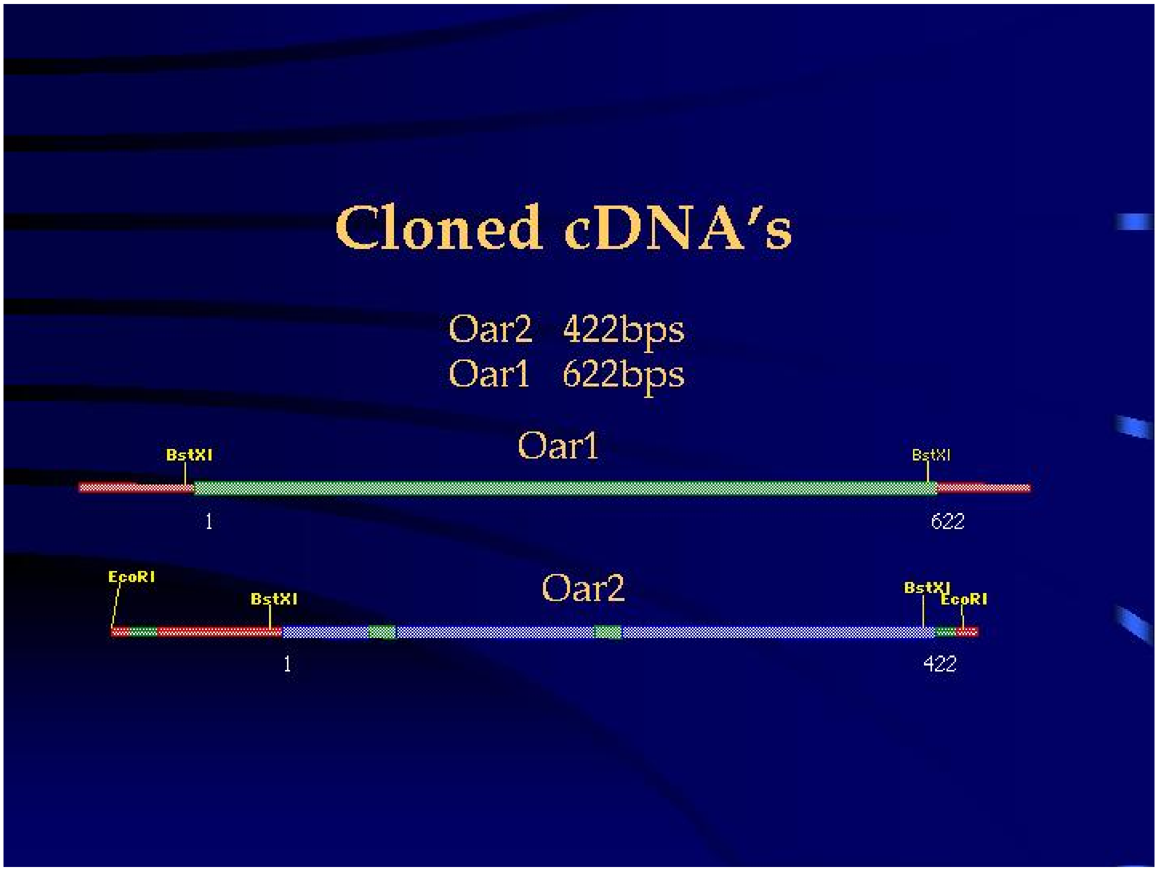

**Fig. 2**

**DISCUSSION.** Isolated cDNAs responsible for Oa resistant affect in OAR1,2 did not point to any known protein, but rather to two recently sequenced genes (AP000795 and AK0021258) of completely unknown function. The elucidation of the importance of the two genes for apoptosis and of how *the* cDNAs of OAR1,2 work, will with high probability shed new light on the mechanisms of OA-induced apoptosis, and is a major undertaking in our laboratory. The novel cell clones will further be used to elucidate the significance for apoptosis of *oar1,2*, and their link to altered protein phosphorylation.

**ACKNOWLEDGEMENT.** This work was funded by the Norwegian Cancer Society.

**REFERENCES.**

1. Cohen, P.T. (1997) Trends Biochem. Sci. 22(7), 245-251
2. Honkanen, R.E. et al. (1994) Toxicon 32(3), 339-350
3. Boe, R. et al. (1991) Exp. Cell Res. 195(1), 237-246
4. Gjertsen, B.T. and Døskeland, S.O. (1995) Biochim. Biophys. Acta 1269(2), 187-199
5. Hitoshi, Y. et al. (1998) Immunity 8(4), 461-471
6. Pear, W.S. et al. (1993) Proc. Natl. Acad. Sci. U S A 90(18), 8392-8396
